# Histone modifications and DNA methylation act cooperatively in regulating symbiosis genes in the sea anemone Aiptasia

**DOI:** 10.1186/s12915-022-01469-y

**Published:** 2022-12-02

**Authors:** Kashif Nawaz, Maha J. Cziesielski, Kiruthiga G. Mariappan, Guoxin Cui, Manuel Aranda

**Affiliations:** 1 Marine Science Program, Biological and Environmental Sciences and Engineering Division, King Abdullah University of Science and Technology (KAUST), Thuwal, Kingdom of Saudi Arabia; 2 Red Sea Research Center, King Abdullah University of Science and Technology, Thuwal, Kingdom of Saudi Arabia

**Keywords:** Symbiosis, Corals, Climate Change, Epigenetics, Histone modifications

## Abstract

**Background:**

The symbiotic relationship between cnidarians and dinoflagellates is one of the most widespread endosymbiosis in our oceans and provides the ecological basis of coral reef ecosystems. Although many studies have been undertaken to unravel the molecular mechanisms underlying these symbioses, we still know little about the epigenetic mechanisms that control the transcriptional responses to symbiosis.

**Results:**

Here, we used the model organism *Exaiptasia diaphana* to study the genome-wide patterns and putative functions of the histone modifications H3K27ac, H3K4me3, H3K9ac, H3K36me3, and H3K27me3 in symbiosis. While we find that their functions are generally conserved, we observed that colocalization of more than one modification and or DNA methylation correlated with significantly higher gene expression, suggesting a cooperative action of histone modifications and DNA methylation in promoting gene expression. Analysis of symbiosis genes revealed that activating histone modifications predominantly associated with symbiosis-induced genes involved in glucose metabolism, nitrogen transport, amino acid biosynthesis, and organism growth while symbiosis-suppressed genes were involved in catabolic processes.

**Conclusions:**

Our results provide new insights into the mechanisms of prominent histone modifications and their interaction with DNA methylation in regulating symbiosis in cnidarians.

**Supplementary Information:**

The online version contains supplementary material available at 10.1186/s12915-022-01469-y.

## Background

Coral reefs are often considered the rainforests of the sea, as they form marine-biodiversity hotspots. Reef ecosystem health directly depends on symbiotic cnidarians, such as corals and anemones, that provide essential habitats for a myriad of marine organisms. To thrive in the oligotrophic environment of tropical oceans, corals, and other symbiotic cnidarians, depend on an intimate endosymbiosis with photosynthetic dinoflagellates of the family Symbiodiniaceae, also known as zooxanthellae [1–3]. Living within the host’s gastrodermal cells, the symbionts provide their hosts with over 90% of their total energy demands [1], making these symbiotic relationships vital for the functioning of the coral reef ecosystem. The disruption of this host-symbiont relationship, also known as bleaching, can result in extensive mortality and subsequent degradation and loss of entire coral reefs [2–4]. Significant efforts have been made to understand the molecular mechanism underlying this relationship [5]. However, there are still substantial knowledge gaps in our understanding of the molecular underpinnings of these relationships, especially pertaining to the role of epigenetic mechanisms in regulating the interactions between the host and the symbionts, which remain elusive in cnidarian symbiosis research [6]. The uptake and maintenance of symbionts require specific host responses, such as the suppression of the immune system [7–10] and the regulation of nutrient fluxes to control symbiont proliferation [11], to maintain a stable symbiotic relationship. Such responses are mediated through transcriptional changes that are known to be regulated via epigenetic mechanisms in other organisms [9, 12–15], and some endosymbionts have even been shown to evoke such responses by directly modifying the epigenome of their hosts [9, 10]. However, while many recent studies have highlighted the importance of epigenetic mechanisms in maintaining symbiotic relationships in plants and animals [16–18], only one study looking at the role of DNA methylation in symbiosis has been conducted in zooxanthellate cnidarians [6, 19].

Eukaryotic genomes are packaged in the form of a DNA-protein complex termed chromatin. The structural subunit of chromatin is known as the nucleosome, which consists of a core protein octamer and a stretch of ~147 bp of DNA that is wound around it. The protein octamer comprises two of each of the core histones H2A, H2B, H3, and H4. Linker histones such as H1 sit externally at the base of nucleosomes, providing further stability to it and contributing to chromatin compaction [20, 21]. This essential organization of histones aids in the folding of the DNA into a higher-order chromatin structures [20–22]. The N-terminal tail of histone proteins can include reversible covalent changes termed post-translational modifications (PTMs), which participate in the formation, folding, and structural/functional regulation of chromatin structure and have thus a profound role in epigenetic regulation of gene expression and genome stability. These PTMs (along with several histone variants) are part of the epigenetic mechanism known as “histone code” [20]. Along with these modifications, other epigenetic mechanisms such as DNA methylation and small RNAs collectively influence the chromatin structure, and consequently, the accessibility of the genetic information [20, 21].

Histone modifications can affect gene regulation differently depending on their type and location in the genome (Table 1). Among the different PTMs, acetylation and methylation of specific histone tail residues have been most extensively studied [12, 13], and they have been found to promote repressing and activating roles in the regulation of gene expression [14]. In general, activator complexes methylate or acetylate-specific amino acid residues in tails of histones bound to gene promoter regions, thereby destabilizing the nucleosome-DNA interaction and facilitating the assembly of the transcriptional machinery at the promoter. However, repressor complexes demethylate/deacetylate histone tails and strengthen the DNA-histone interaction, resulting in hindered accessibility of the respective genomic regions for the transcriptional machinery [15].

**Table 1:** Histone modifications with their function and location

Histone modifications	Function	Location
H3K4me3	Activation	Promoters, bivalent domains
H3K36me3	Activation	Gene body
H3K9ac	Activation	Enhancers, promoters
H3K27ac	Activation	Enhancers, promoters
H3K27me3	Repression	Promoters in gene-rich regions, bivalent domains

Methylation of histone tails occurs mainly at lysine (K) and arginine (R) residues, most commonly observed as mono-, di-, or trimethylation of the lysine residues on H3 and H4 histone tails [23–25]. Methylation of H3K36 and H3K4, for instance, act as activating histone modifications, while H3K27 methylation has a role in repressing the gene expression [26–29]. Similarly, H3K27ac and H3K9ac modifications are associated with active transcription and are predominantly associated with promoter and enhancer regions [26–29]. Histone methyltransferase (HMT) are histone-modifying enzymes that catalyze the transfer of methyl groups to the targeted residues (lysine and arginine) through a domain known as SET domain [30, 31]. Acetylation and deacetylation of histone tails, on the other hand, are catalyzed by histone acetyltransferases (HATs) and histone deacetylases (HDACs), respectively. Different chromatin-modifying enzymes, including histone deacetylases and histone lysine methyltransferases, function through multi-protein complexes that can also interact with methyl-CpG-binding proteins, thereby linking mechanisms of histone modifications to the biochemical mechanism that maintains and modifies DNA methylation [32, 33]. This implies that the enzymatic control of different epigenetic mechanisms is linked via crosstalk, and hence mutually interactive in regulating gene expression [34]. In general, enzymes involved in depositing these chemical modifications (acetyl, methyl, etc.) onto the chromatin at the specific location are known as writers. In contrast, those which remove such modifications are called erasers [35, 36].

Despite the importance of the cnidarian Symbiodiniaceae relationship for ecosystem functioning, we still know very little about the role of epigenetic mechanisms, and specifically histone modifications, in the regulation of host-symbiont interactions. Here, we profiled the genome-wide association of the histone modifications H3K27me3, H3K36me3 and H3K4me, H3K27ac, and H3K9ac, in the cnidarian symbiosis model *Exaiptasia diaphana* (Aiptasia). We describe their genetic context, their correlation with CpG methylation (mCpG), and gene expression, as well as their association with and putative regulation of symbiosis genes.

## Results

### Genome-wide distributions of histone modifications in E. diaphana and their correlation with CpG methylation

To understand the regulatory function of prominent histone modifications and their role in symbiosis, we performed ChIP-seq experiments in symbiotic *E. diaphana* and analyzed the genomic distribution of five major modifications; H3K27me3, H3K36me3, H3K27ac, H3K4me3, and H3K9ac (Additional file 1: Table ST1-ST5), as well as their correlations with respect to DNA methylation [6] and gene expression [11]. To do this, we first called “peaks” from all ChIP-seq data, which are regions in the genome that are actively bound by a respective histone modification (see “Methods” for more details). A circular visualization plot of all five histone modifications with gene and repeat density over the *E. diaphana* genome is shown in Additional file 2: Fig. S1. We used the term “peak” throughout the manuscript to refer to these bound regions as well as their signal intensity relative to the input control.

**Fig. 1:**
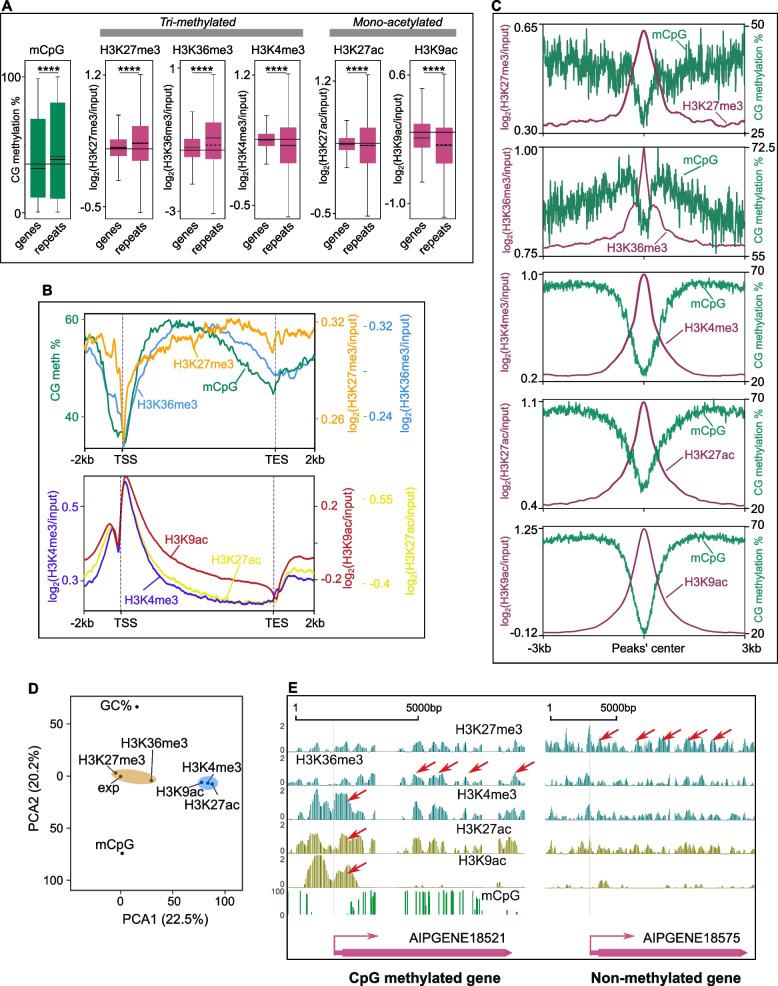
Genome-wide distribution of histone modifications in *E. diaphana* and their correlation with CpG methylation. **A** Boxplots of mCpG, H3K27me3, H3K36me3, H3K4me3, H3K27ac, and H3K9ac peak levels in genes and repeats region of the *E. diaphana* genome. The solid horizontal line in each boxplot represents the median and the dotted line the mean. The solid horizontal line for each modification represents the average median of both genes and repeats (unpaired two-tailed Student’s *t* test; ****p < 0.0001). **B** Enrichment profiles of histone modifications and DNA methylation around all the protein-coding genes. *x*-axis is the gene locations from −2kb of TSS through gene body and +2kb of TES; *y*-axis is the percentage of CpG methylation (mCpG) and log enrichment of peaks for each histone modification. **C** Average mCpG distribution around well-positioned histone modification’s peaks. **D** Principal component analysis of all five histone modifications (H3K27me3, H3K36me3, H3K4me3, H3K27ac, and H3K9ac), CpG methylation (mCpG), GC content (GC %), and gene expression (exp). **E** Genome browser snapshot showing example distribution of all five histone modifications (H3K27me3, H3K36me3, H3K4me3, H3K27ac, and H3K9ac) in gene body and promoter of methylated; AIPGENE18521 and non-methylated; AIPGENE18575 genes. Prominent peaks are labeled with red arrows

For initial validation purposes, we compared the distribution of histone modifications between active and inactive regions of the *E. diaphana* genome [37]. Repeat regions in the genome are mostly silenced [38] and are known to differ in bound histone modifications in comparison to non-repeat, i.e., genic, regions [39]. Genome-wide analysis showed that the modifications H3K27me3 and H3K36me3 had significantly higher peaks (*T*-test; *p <* 0.0001) in repeat regions compared to genic regions. In contrast, H3K4me3, H3K27ac, and H3K9ac had significantly higher peaks (*T*-test; *p <* 0.0001) in genic regions (Fig. 1A). This suggested that the transcriptional suppression of repeat elements in the *E. diaphana* genome aligns with higher H3K27me3 and H3K36me3 signals, and simultaneously lower peak signals for H3K4me3, H3K27ac, and H3K9ac.

Next, we determined the peaks for each histone modification around all protein-coding genes in the *E. diaphana* genome. Peaks of H3K27me3 and H3K36me3 were prevalent in the gene body and promoter regions, but not the transcriptional start site (TSS). In contrast, peaks of H3K4me3, H3K27ac, and H3K9ac exhibited a bimodal peak pattern in the TSS region, with a smaller peak around the promoter region and a prominent peak around the first exon, while gene bodies featured comparatively lower peaks (Fig. 1B, Additional file 2: Fig. S2A and S2B). To investigate the relationship between the different histone modifications and mCpG, we classified all genes from *E. diaphana* as either methylated (*n*=8018) or non-methylated (*n*=21,322) based on their methylation density and methylation level (see “Methods” for more details). We found that H3K27me3 was predominantly present in the gene body of non-methylated genes, while H3K36me3, H3K4me3, H3K27ac, and H3K9ac were associated with methylated genes (adjusted *p* < 0.01) (Additional file 2: Fig. S2C – S2G, Additional file 1: Table ST6).

Interestingly, however, analysis of the core nucleosome regions of all five histone modifications showed either very low or no CpG methylation (Fig. 1C), suggesting that the DNA wound around the histone octamer core containing these modifications is mCpG depleted.

To determine how the different histone modifications are correlated with CpG methylation and gene expression, we analyzed their associations with mCpG, GC content, and gene expression [11] using a principal component analysis (Fig. 1D). All parameters, i.e., histone peak score, mCpG ratio, and GC content, were averaged for each gene. We observed that all histone modifications aligned on the same plane of the second principal component, along with gene expression. This suggests a tighter relationship between histone modifications and gene expressions compared to mCpG or GC content. Conversely, only histone modifications exhibiting gene body prevalence aligned with mCpG, GC content, and gene expression along the first principal component. This is likely because mCpG and GC content also display strong gene body prevalence. Further, gene body prevalent histone modifications (H3K27me3 and H3K36me3) and TSS-prevalent modifications (H3K4me3, H3K27ac, and H3K9ac) clustered together, respectively (Fig. 1D). This suggests that the distribution patterns of mCpG and GC content are more similar to those of gene body prevalent histone modifications. To further confirm this, we performed linear regression analyses to identify potential interactions between histone modifications at each gene (Additional file 2: Fig. S2H – S2K). We found a positive correlation between H3K4me3 and H3K9ac (*R*^*2*^ *= 0.27, p = 0.0023*), H3K27ac and H3K9ac (*R*^*2*^ *= 0.36, p = 0.0014*), and H3K27ac and H3K4me3 (*R*^*2*^ *= 0.43, p = 0.004*), which suggests that a substantial number of genes could potentially be bound by more than one of the three TSS-dominated histone modifications (Additional file 2: Fig. S2H, S2I). In contrast, the correlation between gene body-dominated histone modifications (H3K27me3 and H3K36me3) was very weak (*R*^*2*^ *= 0.052, p = 0.045*) (Additional file 2: Fig. S2J, S2K). Actual examples of the distribution of all five histone modifications over a methylated (AIPGENE18521) and non-methylated (AIPGENE18575) gene are shown in Fig. 1E.

### Histone modification positioning and number as a function of transcription

**Fig. 2:**
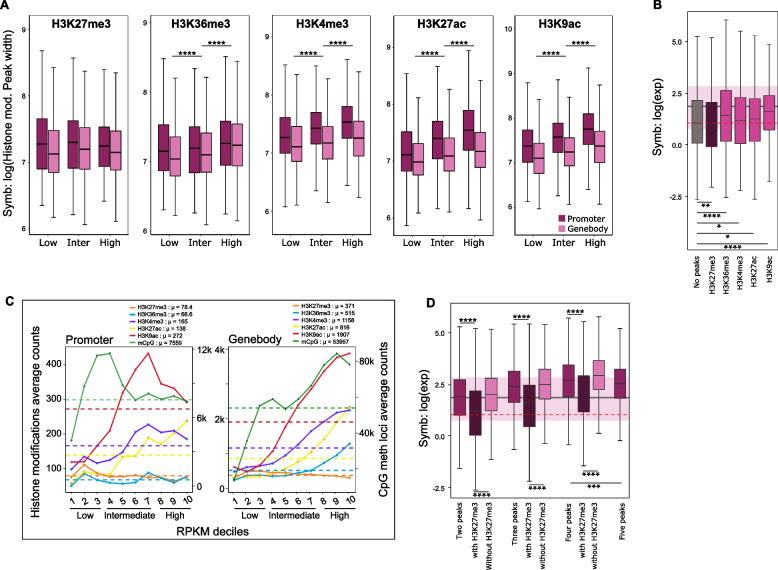
Histone modification positioning and number as a function of transcription. **A** Average peak width of different histone modifications (H3K27me3, H3K36me3, H3K4me3, H3K27ac, and H3K9ac) from promoter and gene body as a function of gene expression category in symbiosis. Magenta boxplots are from promoters and pinks are from gene bodies. *X*-axis is the gene category based on expression level (< 30th percentile RPKM: low, 30th and 70th percentile RPKM: intermediate and > 70th percentile RPKM: high); *y*-axis log value of peaks’ breadth (unpaired two-tailed Student’s *t* test; ****p < 0.0001). **B** Boxplot showing average expression for genes without peaks and genes with peaks for specific histone modifications (unpaired two-tailed Student’s *t* test; *p < 0.05, **p < 0.01, ***p < 0.001, ****p < 0.0001). **C** Specific histone modification counts are represented on the *y*-axis. *X*-axis represents gene expression values binned in deciles according to mRNA abundance (RPKM). Dashed lines represent the average count of each histone modifications and mCpG for all the genes, also shown in *μ*. Different chromatin modifications are represented by colors. **D** Boxplot showing average expression for the genes with two, three, four, and five peaks. And correspondingly with and without the repressive modification H3K27me3 (unpaired two-tailed Student’s *t* test; ***p < 0.001, ****p < 0.0001)

Nucleosome positioning and spacing has previously been shown to correlate with gene expression levels [40]. To study this in *E. diaphana*, we analyzed how the positions of the different histone modifications in the promoter and gene body regions vary and correlate with gene expression. To do this, we used the peak width as a parameter for position, with more precise nucleosome positioning reflecting tighter localization, and hence, narrower peak widths at a given position. For each modification, we compared the average peak width in the promoter and gene body separately (Fig. 2A) across lowly, intermediately, and highly expressed genes. We found that the average peak width of activating histone modifications in the promoter region and the gene body significantly increased with gene expression. Only the repressive modification H3K27me3 did not show a similar trend, and peak width remained constant across all expression levels.

To investigate the effect of multiple peaks on gene expression, we selected genes with no peaks and compared their average expression level against genes with a single peak from one modification. We found that genes that only have a peak for the repressive modification (H3K27me3) exhibited a significantly lower average expression than genes without any peaks. At the same time, all genes with active modifications showed higher gene expression values than genes without any peak or with H3K27me3 (Fig. 2B). Furthermore, we found a general increment in gene expression with an increasing number of peaks for active histone modifications. Meanwhile repressive histone modification peak counts, H3K27me3, showed a negative and weak relation with expression (Fig. 2C). We also examined the cumulative effect of multiple histone modifications on gene expression. Interestingly, we observed that the average gene expression level was higher if genes were associated with more than one histone modification, with every additional modification resulting in significantly higher expression levels of associated genes, as long as the repressive modification H3K27me3 was not included (Fig. 2D). Inclusion of H3K27me3 consistently correlated with significantly lower gene expression levels, further confirming its repressive effect.

### Histone modifications in E. diaphana correlate with gene expression

**Fig. 3:**
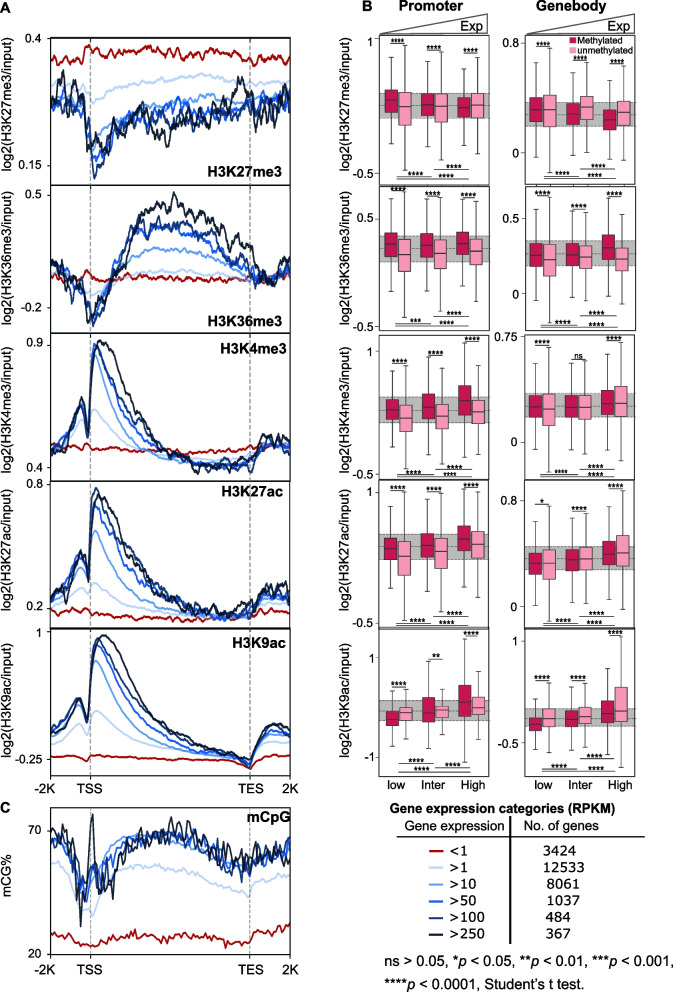
Histone modifications in *E. diaphana* correlate with gene expression. **A** Distribution of histone modifications around genes with increasing expression levels. Red line marking the lowest gene expression category (RPKM < 1), and darkest blue the highest expression category (RPKM >250). **B** Boxplots of histone peak heights from promoter and gene body regions of methylated (dark pink) and unmethylated (light pink) genes. Genes with expression below the 30th percentile of RPKM were classified as lowly expressed, those between the 30th and 70th percentile as intermediately expressed, and those above the 70th percentile as highly expressed. (unpaired two-tailed Student’s *t* test; ∗∗*p <* 0.01, ∗∗∗*p <* 0.001, ∗∗∗∗*p <* 0.0001). **C** mCpG distribution around genes with increasing gene expression levels

To further analyze the correlation of the different histone modifications with mCpG and gene expression, we divided all the protein-coding genes from *E. diaphana* into six categories based on their transcription level and DNA methylation status. We observed a positive correlation between histone peak height and gene expression for H3K36me3 and for all TSS-prevalent histone modification (H3K4me3, H3K27ac, and H3K9ac), and only H3K27me3 displayed a negative correlation (Fig. 3A). This finding affirmed our previous analysis showing that H3K27me3 peak counts decreased with increasing gene expression (Fig. 2C).

To further investigate the potential interactions of histone modifications and DNA methylation, we plotted the average peak heights for every histone modification for low, intermediate, and highly expressed genes, each with and without DNA methylation respectively (Fig. 3B). We found that H3K27me3 showed a significant negative correlation (*p <* 2.2 × 10^−16^) with gene expression both when present in the promoter or the gene body, and this effect was even more pronounced in methylated genes. In contrast, H3K36me3 showed a positive correlation with DNA methylation and gene expression (Fig. 3B), with H3K36me3 peak height positively correlating with increasing expression in methylated genes. Similarly, TSS-prevalent histone modifications, i.e., H3K4me3, H3K27ac, and H3K9ac, also showed a positive correlation with gene expression and methylation (*p <* 2.2 × 10^−16^), and this effect was more pronounced for peaks in the promoter region (Fig. 3A, B).

### Histone modifications regulate the transcriptional response to symbiosis

**Fig. 4:**
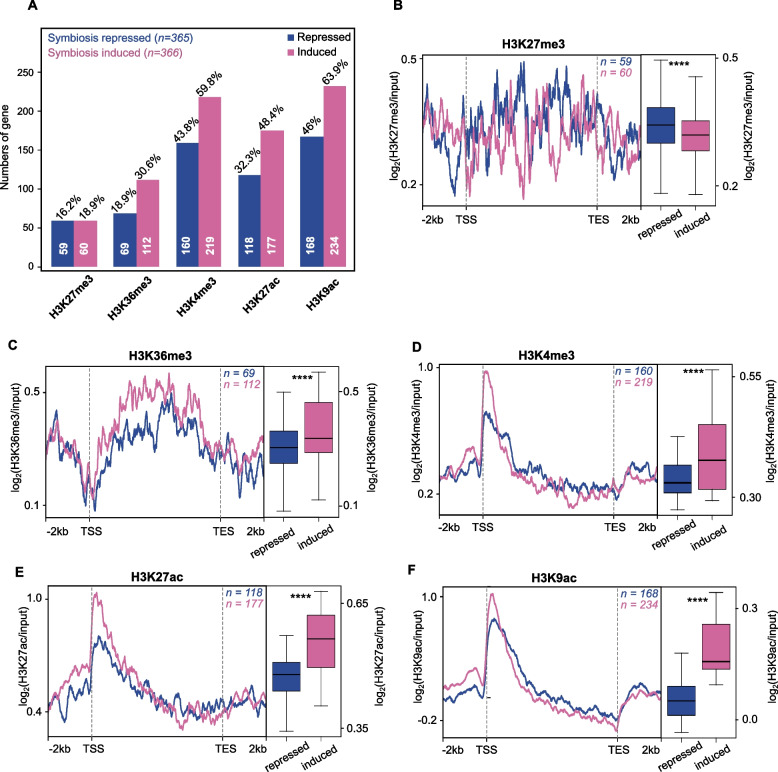
Histone modifications regulate the transcriptional response to symbiosis. **A** Total number of symbiosis-repressed (blue) and induced (pink) genes associated with each histone modification in their gene body and promoter regions. Average peak distributions of symbiosis-repressed (blue) and induced (pink) genes associated with H3K27me3 (**B**), H3K36me3 (**C**), H3K4me3 (**D**), H3K27ac (**E**), and H3K9ac (**F**) from −2kb of TSS through gene body and +2kb of TES. Each of the line plots from symbiosis-repressed genes is compared with induced genes (see respective boxplots, unpaired two-tailed Student’s *t* test; ****p < 2.2 × 10^−16^)

To investigate the role of histone modifications in the regulation of the symbiotic relationship between *E. diaphana* and its dinoflagellate symbionts, we analyzed the correlation between peak occupancy for each histone modification and gene expression across 731 previously identified symbiosis-associated genes [11]. We first categorized these 731 symbiosis-associated genes into symbiosis-repressed (*n*=365) and symbiosis-induced (*n*=366) genes. We found that most symbiosis genes (544; 74.4% of the 731 genes) were associated with at least one of the five histone modifications we analyzed (Fig. 4A, Table 2, Additional file 1: Table ST7 – ST11). Active histone modifications (i.e., H3K36me3, H3K4me3, H3K27ac, and H3K9ac) showed a significantly higher association with symbiosis-induced genes (*p <* 0.01) while the repressive histone modification (H3K27me3) had an almost equal number of peaks in both categories.

**Table 2:** Genes associated with histone modifications in the *E. diaphana* genome and symbiosis genes, respectively

Histone modifications	H3K27me3	H3K36me3	H3K4me3	H3K27ac	H3K9ac	# of genes with at least one modification
**Total in genome**	5660	6536	13,664	9629	14,738	18,675 (63.8%)
**Symbiosis-associated genes**	119 (16.3%)	181 (24.8%)	379 (51.8%)	295 (40.4%)	402 (55%)	544 (74.4%)
**Symbiosis-repressed genes**	59 (16.2%)	69 (18.9%)	160 (43.8%)	118 (32.3%)	168 (46%)	252 (69%)
**Symbiosis-induced genes**	60 (18.9%)	112 (30.6%)	219 (59.8%)	177 (48.4%)	234 (63.9%)	292 (79.8%)

To confirm the relationship between peak height and gene expression, we compared the average histone peak height of every modification across symbiosis-repressed and symbiosis-induced genes. We found that the repressive modification H3K27me3 had significantly higher peaks in symbiosis-repressed genes (*p <* 2.2 × 10^−16^, Fig. 4B), while all active modifications (H3K36me3, H3K4me3, H3K27ac, and H3K9ac) had significantly higher peaks (*p <* 2.2 × 10^−16^) in symbiosis-induced genes (Fig. 4C–F). This finding confirmed that histone modifications play an active role in the regulation of the symbiotic relationship between *E. diaphana* and its dinoflagellate symbiont.

As a final step of validation, we analyzed the correlation between histone modifications and changes in gene expression in response to symbiosis, we divided both symbiosis-induced (*n*=366) and symbiosis-repressed genes (*n*=365) into two groups based on their median gene expression fold change. We compared the profiles of the upper and lower 50th percentile for each of the histone modifications separately. Similar to our previous observation, we found that the repressive modification H3K27me3 showed a higher prevalence in symbiosis-repressed genes irrespective of the fold change (*p <* 2.2 × 10^−16^, Additional file 2: Fig. S3A). Conversely, our analysis on the active histone modifications; H3K36me3, H3K4me3, H3K27ac, and H3K9ac, also confirmed our previous findings of significantly higher peaks in symbiosis-induced genes (*p <* 2.2 × 10^−16^) (Additional file 2: Fig. S3B – S3E).

### Histone modifications are involved in symbiosis-induced nutrient metabolism

To understand the role of histone modifications in the regulation of the symbiotic relationship, we performed gene ontology (GO) enrichment analyses for both symbiosis-repressed and symbiosis-induced genes for each histone modification individually (Additional file 1: Table ST12 – ST21, Additional file 2: Fig. S4). While each modification had several unique enriched biological functions, we found a considerable number of categories that were enriched across two or more histone modifications (Additional file 1: Table ST22 – ST23). Many of these shared categories within the symbiosis-induced genes were involved in amino acid metabolic processes, such as the regulation of cellular amino acid and protein metabolic process (Table 3). Furthermore, we found processes involved in the response to glucose and ammonium transport to be associated with multiple modifications, suggesting that the central function of this symbiotic relationship in driving host amino acid biosynthesis is regulated through histone modifications [11]. In line with this, we also found shared enrichment of amino acid biosynthesis-related processes, including L-serine, glutamine, and methionine, as well as categories involved in amino acid transport. Similarly, we found several shared biological processes associated with organism growth to be enriched across multiple histone modifications, most notably GO terms related to central growth pathways such as the insulin, hippo, and the TORC1 pathways.

**Table 3:** Selected GO terms of symbiosis genes associated with histone modifications. The full GO term list is shown in Additional file 1: Tables (ST12 – ST23)

GO term	Description
GO:0000098	Sulfur amino acid catabolic process
GO:0003333	Amino acid transmembrane transport
GO:0006521	Regulation of cellular amino acid metabolic process
GO:0006527	Arginine catabolic process
GO:0006541	Glutamine metabolic process
GO:0006559	L-phenylalanine catabolic process
GO:0006564	L-serine biosynthesis process
GO:0006579	Amino acid betaine catabolic process
GO:0009086	Methionine biosynthesis process
GO:0009115	Xanthine catabolic process
GO:0009749	Response to glucose stimulus
GO:0015804	Neutral amino acid transport
GO:0019518	L-threonine catabolic process to glycine
GO:0031931	TORC 1 complex
GO:0032024	Positive regulation of insulin secretion
GO:0035329	Hippo signaling pathway
GO:0044267	Cellular protein metabolic process
GO:0046168	Glycerol-3-phosphate catabolic process
GO:0072488	Ammonium transport
GO:0046949	Fatty-acyl-CoA biosynthetic process
GO:0046500	S-adenosylmethionine metabolic process
GO:0006556	S-adenosylmethionine biosynthetic process
GO:0008898	S-adenosylmethionine-homocysteine S-methyltransferase activity
GO:0032259	Methylation
GO:0001733	Galactosylceramide sulfotransferase activity
GO:0003943	N-acetylgalactosamine-4-sulfatase activity
GO:0003810	Protein-glutamine gamma-glutamyltransferase activity
GO:0070403	NAD+ binding
GO:0004029	Aldehyde dehydrogenase (NAD+) activity

While many of the enriched categories in the symbiosis-induced genes were associated with anabolic processes, we found symbiosis-repressed genes associated with histone modifications to be predominantly involved in catabolic processes. These included the catabolism of molecules like xanthine and glycerol-3-phosphate but also amino acids such as sulfur amino acids, L-phenylalanine, betaine, arginine, and L-threonine, among others.

Interestingly, we also found enrichment of GO terms involved in various metabolic pathways that generate metabolites important for epigenetic modifications, such as acetyl-CoA/fatty-acyl-CoA, S-adenosylmethionine, methylation process, and lactate. Previous studies have shown that these metabolites serve as cofactors for the enzymes responsible for depositing the chemical modifications (acetyl and methyl) onto chromatin; chromatin writers [35, 36]. In addition, we found GO terms related to metabolites such as α-ketoglutarate and NAD+, which are essential cofactors for certain enzymes that remove chemical modifications; chromatin erasers [35, 36]. This suggests that the chromatin changes induced to regulate gene expression in response to symbiosis might be supplied by these processes and ultimately established through the respective writers and erasers.

## Discussion

The process of symbiosis establishment and maintenance requires changes in the cnidarian host’s cell function and specialization. Epigenetic mechanisms have been shown to play critical roles in symbiotic relationships of eukaryotic and bacterial cells [16]. The general importance of histone modifications in host-microbe interactions has been acknowledged in plants, humans, and other invertebrates [16–18]. Through chemical signals and metabolites, endosymbionts can influence epigenomes of host cells and directly enable communication between the two partners [18, 41]. Interestingly, histone acetylase and deacetylase activity have been shown to be influenced by microbes and dietary factors [10, 42, 43]. Although epigenetic studies in cnidarians remain scarce, there is evidence that histone modifications may play a critical role in host-algae symbiosis mechanisms [6, 44–46]. Here, we report the first genomic landscape of five histone modifications, H3K27me3, H3K36me3, H3K4me3, H3K27ac, and H3K9ac, in a symbiotic cnidarian.

We find that their genomic distribution and putative primary functions align with observations made in other organisms [47], suggesting functional conservation of these histone modifications in *E. diaphana*. Further, our results revealed strong correlations between the histone modifications analyzed and transcriptional changes observed in response symbiosis. These findings collectively suggest a direct role for histone modifications in regulating the host’s response to symbiosis.

### Conserved roles of histone modifications in regulating gene expression

The general explanation for the ability of histone modifications to enhance or repress transcription is that they affect the DNA-histone association and, thus, promote or suppress access for transcription factors and the transcriptional machinery to the DNA. As such, these modifications represent an essential mechanism for the epigenetic control of transcriptional responses in eukaryotes [13, 48–50]. In line with this, our analyses revealed highly significant correlations between histone modifications and gene expression. Analysis of the genomic distribution of H3K27me3 and H3K36me3 showed enrichment in repeat regions [48, 49] while the activating modifications H3K4me3, H3K27ac, and H3K9ac showed enrichment in the genic regions (Fig. 1A), as expected based on observations in other organisms [12, 51].

It is interesting to note, however, that we found a substantial number of genes associated with more than one histone modification, suggesting that several histone modifications might act on the same gene simultaneously (Additional file 2: Fig. S2H – S2K). Such a cooperative interaction in regulating gene expression was further supported by the finding that the number of active histone modifications present on genes positively correlated with gene expression levels, suggesting an additive effect. However, it needs to be pointed out that ChIP-seq data cannot inform if the modifications were present on the same DNA molecule or if they were just associated with the same gene but in different cells of the organism. This limitation is evident when looking at the lower average expression observed for genes that were bound by an activating histone modification as well as the repressive modification H3K27me3. Since an actively expressed gene is unlikely to be simultaneously associated with activating and repressive modification, it is more likely that the H3K27me3 association stems from cells where this gene was silenced. Since these cells would not contribute any transcripts for this gene to the whole organism RNA pool, this would reduce the observed overall expression level of the gene in the organism.

### Crosstalk between histone code and DNA methylation

DNA has a determined nucleotide sequence that cannot be changed. However, it has been postulated that the transcription of the genetic information is partly regulated by epigenetic mechanisms such as the underlying histone modifications and DNA methylation. Our analyses of potential interactions of histone modifications and DNA methylation in regulating gene expression revealed strong correlations for all activating modifications that suggest crosstalk between these epigenetic mechanisms in *E. diaphana*. We observed that the average expression of genes associated with activating histone modifications was generally higher if they were also methylated (Fig. 3B), suggesting a cooperative interaction between activating histone modifications and DNA methylation. In contrast, we found that genes associated with the repressive modification H3K27me3 showed the opposite trend for methylated genes if the histone modification was found in the gene body. However, it should be noted that H3K27me3 was predominantly present in the gene body of non-methylated genes, while H3K36me3, H3K4me3, H3K27ac, and H3K9ac were associated with methylated genes (Additional file 2: Fig. S2C – S2G). While these results suggested a cooperative interaction, analysis of the core nucleosome regions of all five histone modifications showed either very low or no CpG methylation (Fig. 1C), indicating that they are present on the same genes but that their precise locations within the gene are mutually exclusive. In summary, our results are indicative of crosstalk between active histone modifications and DNA methylation in modulating gene expression, while repressive modifications associate predominantly with non-methylated genes to suppress their expression.

### A model for the regulation of gene expression via histone modifications in E. diaphana

Based on our results, we propose a model for how the histone modifications analyzed here could regulate gene expression in *E. diaphana*. In addition, the model demonstrates how histone modification and DNA methylation crosstalk may be functioning in symbiotic cnidarians.

**Fig. 5:**
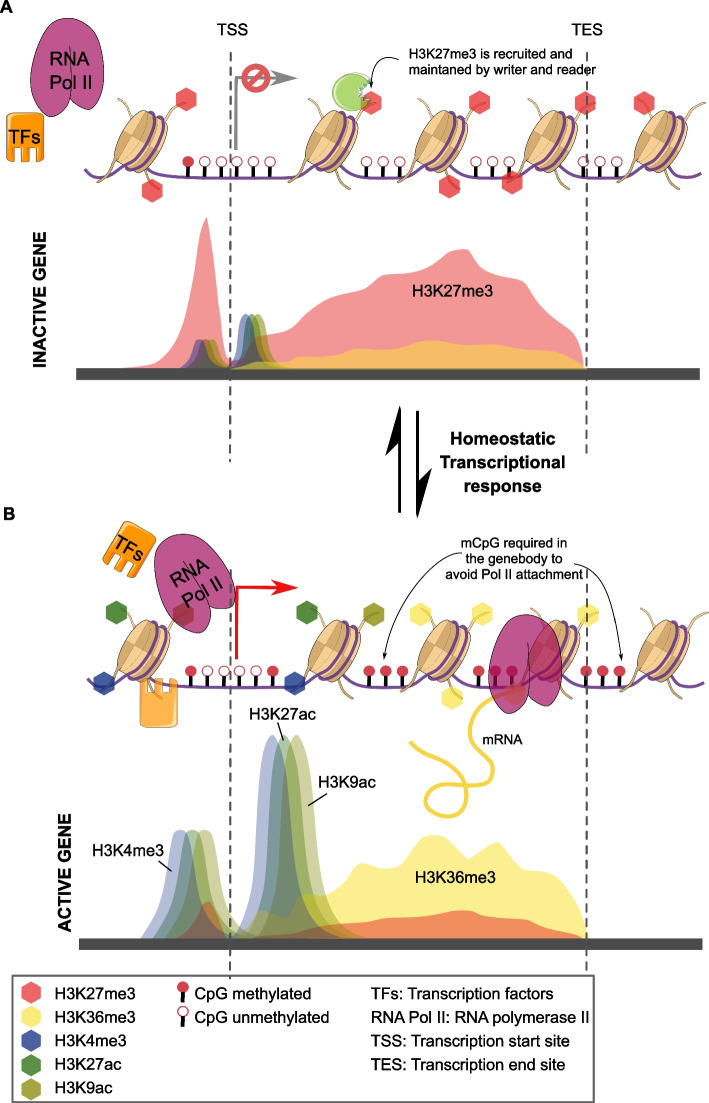
Histone modifications and CpG methylation underlying dynamic gene regulation. Proposed model for the dynamic topology of five histone modifications and CpG methylation on gene loci: **A** Chromatin erasers remove all active histone modifications and DNA methylation from the gene body (H3K36me3 and CpG) and promoter (H3K4me3, H3K27ac, H3K9ac, and CpG) of an inactivated gene. Simultaneously, chromatin writers add the repressive mark H3K27me3 to histone H3 molecules within the promoter and gene body. This chromatin state prevents RNA Pol II and transcription factors (TFs) from attaching to the promoter. **B** For the activation of gene expression H3K4me3, H3K27ac, and H3K9ac are established at the promoter and the transcriptional start sites of the gene, while H3K36me3 and CpG methylation are established throughout the gene body. This facilitates access of Pol II and TFs to the promoter and the TSS, which activates the gene and promotes transcription

When a gene is silenced, it is bound by H3K27me3 in the promoter and gene body (Fig. 5A), which promotes repression through the polycomb complex. H3K27me3 has been shown to recruit PRC1 (polycomb repressive complex), which contributes to the compaction of the chromatin, leading to the formation of heterochromatin and its inaccessibility for transcription factors and the transcriptional machinery [52].

However, when a gene needs to be activated, it requires the preinitiation complex (PIC) to assemble at the promoter region of a gene and to recruit RNA Pol II to the promoter to build the transcription initiation complex [53–55]. Based on our results, and in line with previous findings [12, 28, 56], we propose that the presence of the activating histone modifications H3K27ac and H3K9ac around the promoter and the TSS promote access for transcription factors of the PIC to the gene promoter (Fig. 5B). Once the PIC is assembled, RNA Pol II can be recruited to form the transcription initiation complex and H3K27ac, H3K9ac, and H3K4me3 (TSS-dominated modifications) can act as a pause-release signal for Pol II to initiate transcription. The transcriptional elongation process is then supported by H3K4me3 and H3K36me3 [57]. Meanwhile, low CpG methylation at the promoter further favors the attachment of the assembly of the PIC and the transcriptional complex [58], while high CpG methylation in the gene body prevents the assembly of the transcriptional machinery at cryptic promoter sequences within the gene body, which would lead to spurious transcripts and the production of truncated proteins [59]. The crosstalk between histone modifications and DNA methylation is brought about through the interaction of histone-modifying enzymes. For instance, the histone methyltransferase Set2D is recruited by the active transcriptional complex and trimethylates H3K36 along the gene body. H3K36me3, in turn, is then actively bound by DNA methyltransferase 3b which methylated CpG within the gene body of actively transcribed genes [60]. Together, histone modifications and DNA methylation create a chromatin landscape conducive of high gene expression and the production of full-length transcripts, while at the same time reducing transcriptional noise and spurious transcripts (Fig. 5B) [61].

### The role of histone modifications in symbiosis

Our analyses revealed that genes associated with activating histone modifications were significantly enriched in the fraction of symbiosis-induced genes. This suggests that their increased expression in response to symbiosis is promoted via their association with activating histone modifications. Interestingly, we did not see the opposite trend for the repressive modification H3K27me3 which is associated with the same number of symbioses induced and repressed genes. However, analysis of H3K27me3 peak heights did show significantly higher peaks in symbiosis-repressed genes compared to symbiosis-induced ones. The fact that H3K27me3 peaks were significantly higher in symbiosis-repressed genes suggests that the association of these genes with H3K27me3 was evident in more host cells, which increased the number of ChIP-seq reads obtained, and thus the relative peak heights.

Analyses of the biological functions enriched in 2 or more histone modifications highlighted that the histone modifications studied associated with anabolic functions in symbiosis-induced genes and catabolic functions in symbiosis-repressed genes. In particular, processes associated with amino acid biosynthesis and growth suggested that these histone modifications are involved in regulating the metabolic response and growth in symbiotic anemones. However, these processes are also involved in the regulation of the symbiotic relationship itself. Endosymbiotic relationships are usually driven by synergies arising from the complementation of the host’s metabolic capabilities that enable the resulting metaorganism to thrive in nutrient-poor environments or to use previously inaccessible diets [62, 63], as is also the case for symbiotic anemones and corals. However, this intimate form of symbiosis requires maintaining a delicate balance of nutrient fluxes to provide nutrients to the symbionts, to keep benefiting from them, but at the same time ensure they do not over proliferate at the expense of the host. The maintenance of this balance in symbiotic cnidarians is achieved through the regulation of genes involved in ammonium assimilation and amino acid biosynthesis [11]. We find that genes involved in the assimilation of waste ammonium and amino acid biosynthesis are predominantly associated with activating histone modifications. Further, the observed crosstalk of activating histone modifications and DNA methylation in driving higher expression of symbiosis-induced genes suggest a multi-layer epigenetic regulatory mechanism that may be critical for cnidarian symbiosis.

## Conclusions

In summary, our analysis of the genome-wide distribution of five histone modifications in the symbiotic sea anemone Aiptasia identified generally conserved patterns for all the modifications. Correlations of histone modifications, DNA methylation patterns, and gene expression further suggest a cooperative function of histone modifications and DNA methylation in regulating gene expression. Analysis of previously identified symbiosis genes revealed a functionally consistent association with active and repressive histone modifications and DNA methylation, indicating that the maintenance of symbiosis-associated gene expression is likely mediated by the synchronous action of both epigenetic mechanisms. However, the biological interpretations of the results presented here are only first insights into research that clearly requires further expansion. We acknowledge that to further decipher the role of histone modifications in symbiosis, more ChIP-seq studies including aposymbiotic individuals (*E. diaphana* without its dinoflagellate symbionts) will be required. Nonetheless, our results on the biological functions align with recent observations of regulated gene accessibility and chromatin states in symbiotic anemones [44].

## Methods

### E. diaphana culture and maintenance

*E. diaphana* of the clonal strain CC7 [64], originating from North Carolina, was used in this study. Anemones were maintained in polycarbonate tubs with autoclaved seawater at 25°C. Animals were exposed for 12-h light/dark cycle at 20–40 μmol photons m^2^s^1^ light intensity. The anemones were fed twice weekly with freshly hatched *Artemia nauplii* (brine shrimp). For the experiment, three independent biological replicates were taken, each consisting of two individual anemones to provide enough material for all immunoprecipitations. Each of the three biological replicates was then processed independently for chromatin extraction, resulting in 3 independent chromatin samples.

### Chromatin immunoprecipitation (ChIP) sequencing library preparation

The process of establishing a reproducible ChIP-seq protocol in *E. diaphana*, which so far has primarily been optimized for human, mice, and plant cell studies, included many quality control and optimization steps that require attention. In hopes of streamlining future attempts at ChIP-seq in other cnidarians, especially corals, we opted to optimize pre-immunoprecipitation steps to the point that kits could be confidently used thereafter. We used Zymo-Spin ChIP Kit (Zymo Research) to extract histone-bound DNA fragments; however, we applied minor adjustments to the pre-IP steps. In a recent publication [6], we published a summarized version of the protocol. In another publication [65], a summarized version of the protocol was presented. Here, we provide a detailed description of the protocol (see also Additional file 2); steps of validation and optimization are described in greater detail to, hopefully, allow future research to progress and further advance the field of epigenetic research in cnidarians.

Corresponding input controls for each of the three replicates were generated as suggested by the manufacturer. After validation of various histone antibodies (Additional file 2: Fig. S4), immunoprecipitation was conducted using a target-specific antibody to histone 3 acetylation at lysine 27 – H3K27ac (ab4729, Abcam), histone 3 trimethylation at lysine 4 – H3K4me3 (ab8580, Abcam), histone 3 acetylation at lysine 9 – H3K9ac (ab10812, Abcam), histone 3 trimethylation at lysine 36 – H3K36me3 (ab9050, Abcam), and histone 3 trimethylation at lysine 27 – H3K27me3 (ab6002, Abcam). Each of the three independent chromatin extractions (biological replicates) was divided into 6 aliquots to perform immunoprecipitations for each of the 5 histone modifications as well as retaining an input control for each biological replicate. This resulted in 15 immunoprecipitations and 3 input controls, one for each biological replicate. Upon validation of immunoprecipitations, using High Sensitivity DNA Reagents (Agilent Technologies, California, USA) on a Bioanalyzer, ChIP libraries were constructed using TruSeq Nano HT DNA kit (Illumina, California, USA).

### Sequencing libraries

Paired and single-end sequencing was performed for all 24 libraries at the Bioscience Core Lab (BLC) at the King Abdullah University of Science and Technology, Thuwal, KSA, with NextSeq 500. The ChIP-seq mapped files are deposited in NCBI SRA under accession number PRJNA826667.

### Sequence alignments

ChIP-seq library sequencing resulted in 10 to 40 million single- and paired-end reads per replicate. Reads from 24 libraries (3 biological replicates for each of the 5 histone modifications; upon which 3 (H3K27ac, H3K27me3, and H3K4me3) modifications have common input controls library while 2 (H3K9ac and H3K36me3) have separate input controls) were checked for their quality with FASTQC toolkit [66]. Adapters were removed and reads were cleaned using Trimmomatic [67]. Subsequently, clean reads were uniquely mapped on the *E. diaphana* genome (http://Exaiptasia diaphana.reefgenomics.org/) [37] using bowtie 1.1.2 with default parameters [68].

### Identification and annotation of histone modification peaks

Bam files resulting from the mapping of the 24 libraries were used to call histone peaks separately for each modification using the Model-based Analysis of ChIP-Seq (MACS3: https://github.com/macs3-project/MACS/tree/master/MACS3) [69]. Genomic regions for the five different modifications (3 replicates each) were identified through “*macs3 callpeak -t treatment.bam -i input.bam -f BAM -g 2.7e*^*+8*^ *-B --nomodel --d-min 10 --call-summits*” parameters. Subsequently, the peak output of the replicates from each sample were combined for a replicate aware analysis using the Multiple Sample Peak Calling tool (MSPC) (https://github.com/Genometric/MSPC) [70], with “./mspc -I rep*.bed -r bio -w 1e-4 -s 1e^*-8*^” parameters. Two filters were applied for final peak calling; (1) in MACS3, we filtered each called peak by adjusted *p* < 0.01, and (2), in MSPC, consensus regions of peaks from MACS3 were called across the three replicates of each histone modification with adjusted *p* < 0.01. MSPC generated a single file from the three biological replicates with genome-wide peaks for each histone modification, which was used for all downstream analyses.

Each MSPC generated histone enrichment file with peak locations was annotated based on the *E. diaphana* genome (GFF3 file) [37] using ChIPseeker: An R/Bioconductor package for ChIP peak annotation, comparison, and visualization [71]. This package annotates peaks based on genome features, e.g., genic or intergenic region, as well as distances to the 5′ and 3′ ends of each genomic feature (promoter/exon/intron). The annotated tables of all five histone enrichment peaks are provided in Additional file 1: Tables ST1–ST5.

### Analysis of previously published RNA-seq and DNA methylation data

Gene expression data were obtained from a previously published study in our lab [11]. This data was generated through a meta-analysis with random effects across four independent differential gene expression data sets. We identified a robust set of 731 genes involved in symbiosis (Additional file 1: Table ST24). Based on the median gene expression fold change, we classified these 731 genes into symbiosis-induced (*n*=366) and symbiosis-repressed (*n*=365) genes.

For DNA methylation, we used BS-seq raw reads from symbiotic *E. diaphana* previously published by Li et al. [6]. We used the six replicates from the symbiotic condition to determine the methylation level following the steps described in Li et al. [6]. After filtering the reads for their quality, we first mapped the reads to *E. diaphana* genome with bowtie 1.1.2 [68] and performed methylation calling using Bismark-0.22.3 [72]. We applied three filters to reduce false positives as mentioned in Li et al. [6] and annotated methylated locations using ChIPseeker.

### GO enrichment of histone peaks

To analyze the functional enrichment of the histone peak-bound genes, we obtained the GO annotation from the genome annotation and analyzed it using topGO [73] using default settings. In order to test for the potential role of all five histone modifications in symbiosis, we used the list of 731 identified symbiosis genes as identified in Cui et al. [8] and matched them with the binding sites of each histone modification.

### Data visualization

Average enrichment scores, plots, and heatmaps at genomic features of interest were generated using deepTools [74]. Histone peak width and read counts for each associated gene and modification were determined and plotted in boxplots, and statistical tests to compare the means of two or more groups were performed in R (version 3.5.1 and R CRAN package: *dyplr*). In boxplots, the bottom and top of the box indicate the 25th and 75th percentile, respectively. The horizontal thick bars in the boxplot denote the medians. Whiskers indicate the 1.5X interquartile range (IQR).

Integrated Genome Browser - 9.1.8 (IGB) [75] has been used to explore and visually analyze different histone modification peaks and DNA methylation on the *E. diaphana* genome. Circular visualization plots were generated in R by circlize package [76].

## Supplementary Information

**Additional file 1: Supplementary Table ST1.** Description of all the peaks estimated from three replicates for H3K27me3, using Multiple Sample Peak Calling (MSPC) program. **Supplementary Table ST2.** Description of all the peaks estimated from three replicates for H3K36me3, using Multiple Sample Peak Calling (MSPC) program. **Supplementary Table ST3.** Description of all the peaks estimated from three replicates for H3K4me3, using Multiple Sample Peak Calling (MSPC) program. **Supplementary Table ST4.** Description of all the peaks estimated from three replicates for H3K27ac, using Multiple Sample Peak Calling (MSPC) program. **Supplementary Table ST5.** Description of all the peaks estimated from three replicates for H3K9ac, using Multiple Sample Peak Calling (MSPC) program. **Supplementary Table ST6.** Description of all the peaks estimated from three replicates for each histone modification, using Multiple Sample Peak Calling (MSPC) program. **Supplementary Table ST7.** List of Symbiosis-repressed and -induced genes with H3K27me3 peaks. **Supplementary Table ST8.** List of Symbiosis-repressed and -induced genes with H3K36me3 peaks. **Supplementary Table ST9.** List of Symbiosis-repressed and -induced genes with H3K4me3 peaks. **Supplementary Table ST10.** List of Symbiosis-repressed and -induced genes with H3K27ac peaks. **Supplementary Table ST11.** List of Symbiosis-repressed and -induced genes with H3K9ac peaks. **Supplementary Table ST12.** GO IDs and terms of symbiosis induced genes associated with H3K27me3. **Supplementary Table ST13.** GO IDs and terms of symbiosis repressed genes associated with H3K27me3. **Supplementary Table ST14.** GO IDs and terms of symbiosis induced genes associated with H3K36me3. **Supplementary Table ST15.** GO IDs and terms of symbiosis repressed genes associated with H3K36me3. **Supplementary Table ST16.** GO IDs and terms of symbiosis induced genes associated with H3K4me3. **Supplementary Table ST17.** GO IDs and terms of symbiosis repressed genes associated with H3K4me3. **Supplementary Table ST18.** GO IDs and terms of symbiosis induced genes associated with H3K27ac. **Supplementary Table ST19.** GO IDs and terms of symbiosis repressed genes associated with H3K27ac. **Supplementary Table ST20.** GO IDs and terms of symbiosis induced genes associated with H3K9ac. **Supplementary Table ST21.** GO IDs and terms of symbiosis repressed genes associated with H3K9ac. **Supplementary Table ST22.** GO IDs and terms of symbiosis induced genes associated with one or more histone marks. **Supplementary Table ST23.** GO IDs and terms of symbiosis repressed genes associated with one or more histone marks. **Supplementary Table ST24.** List of all symbiosis induced and repressed genes list from Cui *et al.,* 2019.

**Additional file 2: Figure S1.** Circular visualization of histone modifications, gene and repeat density over the 7 largest scaffolds of *E. diaphana* genome. **Figure S2.** Genome-wide distribution of histone modifications in *E. diaphana* and their correlations. **Figure S3.** Upper and lower 50% percentile: histone modifications change within symbiosis induced and repressed genes. **Figure S4.** Gene Ontology bubble plots. **Figure S5.** Sequences alignment of histone 3 (H3) across species. **Figure S6.** Western blot of histone specific antibodies on total protein content of Aiptasia. **Table S1.** Table showing fixation buffer chemicals and its concentrations. **Table S2:** Table showing nucleic preparation buffer chemicals and its concentrations. **Figure S7.** Schematic representation of ChIP-seq protocol using Aiptasia.

## Data Availability

The ChIP-seq mapped files generated for this study have been submitted to the NCBI Sequence Read Archive (https://www.ncbi.nlm.nih.gov/sra/?term=PRJNA826667) under accession number PRJNA826667 [6,11,77]. All other data are available as indicated in the main text and the supplementary materials.
