# Supplementary material for: Histone modifications and DNA methylation act cooperatively in regulating symbiosis genes in the sea anemone Aiptasia
Source: BMC Biol. 2022 Dec 2;20:265. doi: 10.1186/s12915-022-01469-y (PMC9717517; doi:10.1186/s12915-022-01469-y)
Supplement: Supplementary file 2 — Additional file 2: Figure S1. Circular visualization of histone modifications, gene and repeat density over the 7 largest scaffolds of E. diaphana genome. Figure S2. Genome-wide distribution of histone modifications in E. diaphana and their correlations. Figure S3. Upper and lower 50% percentile: histone modifications change within symbiosis induced and repressed genes. Figure S4. Gene Ontology bubble plots. Figure S5. Sequences alignment of histone 3 (H3) across species. Figure S6. Western blot of histone specific antibodies on total protein content of Aiptasia. Table S1. Table showing fixation buffer chemicals and its concentrations. Table S2: Table showing nucleic preparation buffer chemicals and its concentrations. Figure S7. Schematic representation of ChIP-seq protocol using Aiptasia. [file 12915_2022_1469_MOESM2_ESM.docx]

Supplement: Additional File 2:Fig S1-S7, Table S1-S2
Histone modifications and DNA methylation act cooperatively in regulating symbiosis genes in the sea anemone Aiptasia.

**Authors**

Kashif Nawaz^1,2†*^, Maha J. Cziesielski^1,2†^, Kiruthiga G. Mariappan^1,2^, Guoxin Cui^1,2^, Manuel Aranda^1,2*^

^†^These authors contributed equally.

^*^Corresponding authors. Email: [kashif.nawaz@kaust.edu.s](mailto:kashif.nawaz@kaust.edu.s)a and [manuel.aranda@kaust.edu.sa](mailto:manuel.aranda@kaust.edu.sa)


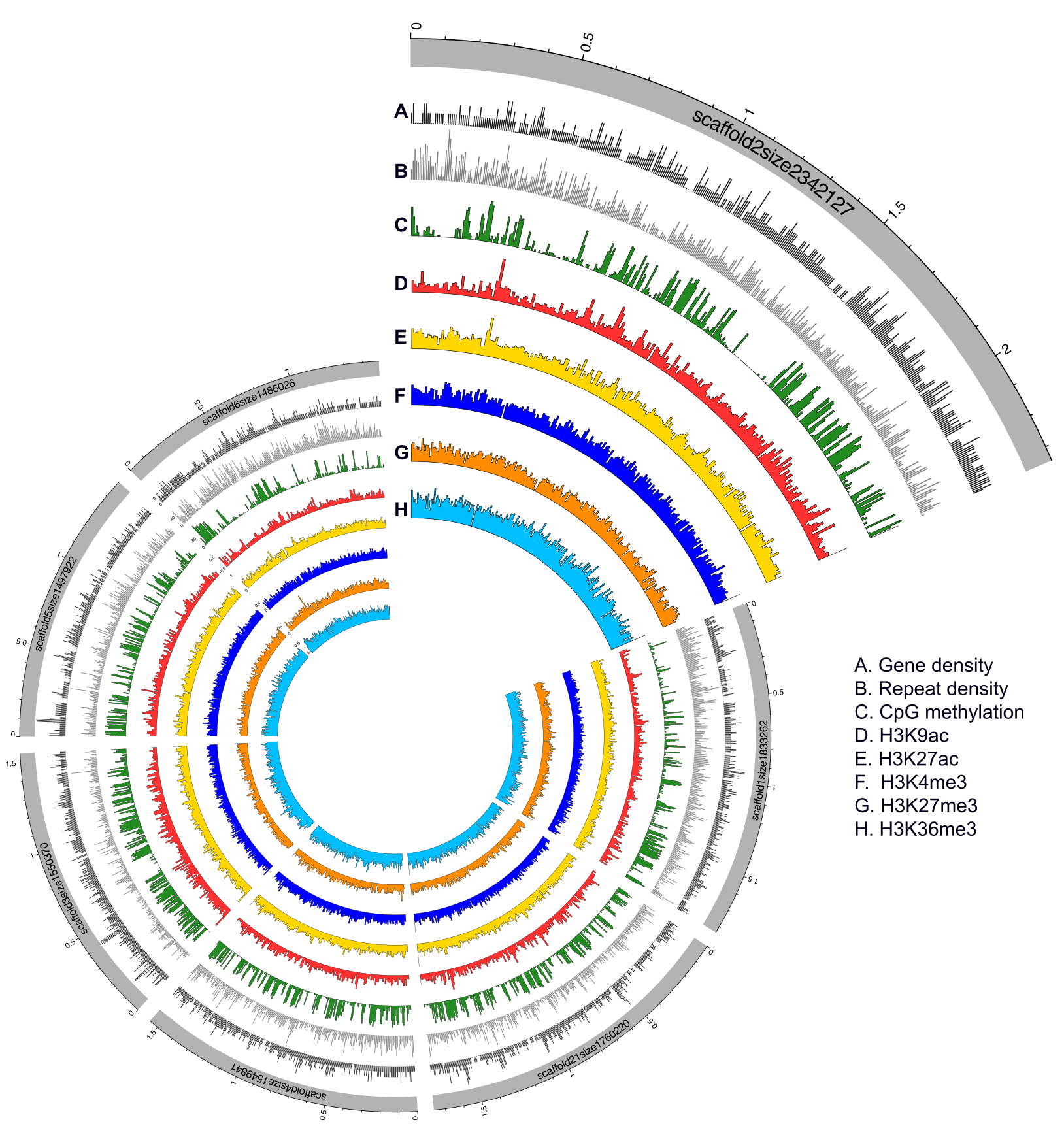


**Supplementary Figure S1.** Circular visualization of gene density **(A)**, repeat density **(B)**, CpG methylation **(C)**, H3K9ac **(D)**, H3K27ac **(E)**, H3K4me4 **(F)**, H3K27me3 **(G)** and H3K36me3 **(H)** over the 7 largest scaffolds of the *E. diaphana* genome assembly.


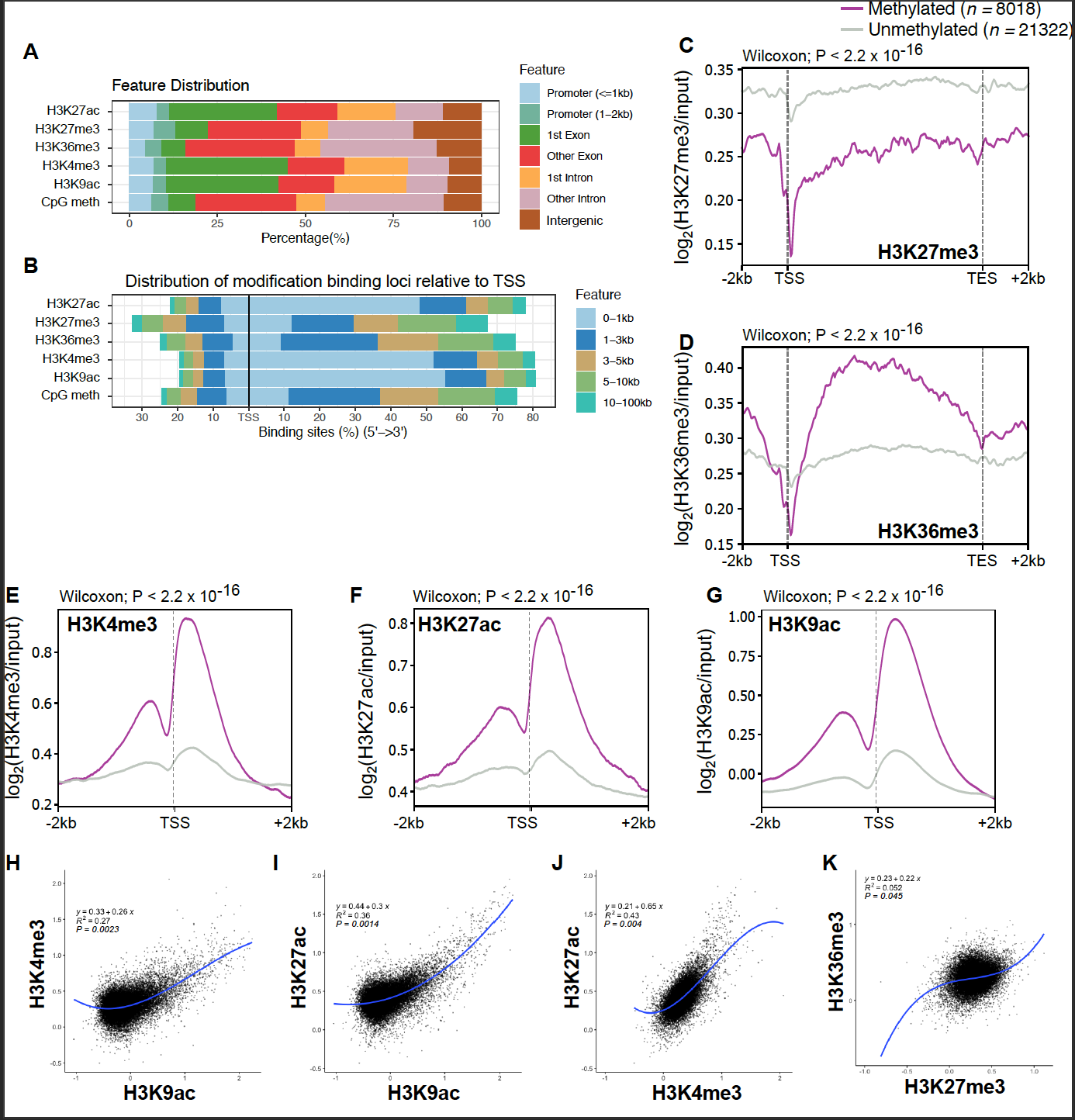


**Supplementary Figure S2.** **Genome-wide histone modifications distributions in *E. diaphana* and their Correlations.**

**(A)** Genomic distribution of significantly identified peaks from different histone modifications and mCpG in Aiptasia genome.

**(B)** Distribution of significantly identified peaks from different histone modifications and mCpG with respect to TSS in Aiptasia genome.

Average peaks of methylated (pink) and unmethylated (grey) genes associated with H3K27me3 **(C)**, H3K36me3 **(D)**, H3K4me3 **(E)**, H3K27ac **(F)** and H3K9ac **(G)** from -2kb of TSS through gene-body and +2kb of TES.

Linear regression analyses between H3K9ac with H3K4me3 **(H)**, H3K9ac with H3K27ac **(I)**, H3K4me3 with H3K27ac **(J)**, and H3K27me3 with H3K36me3 **(K)**.


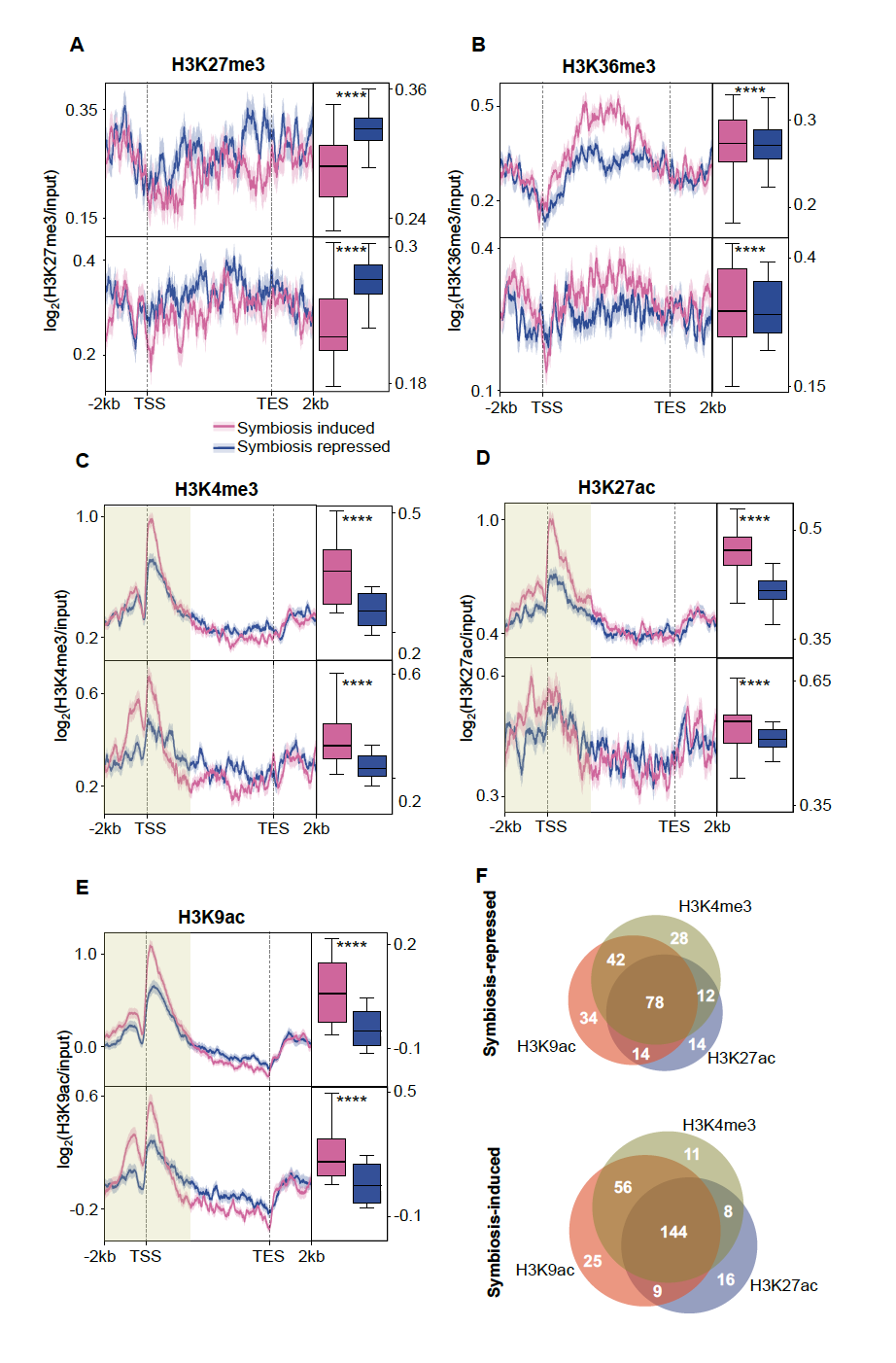


**Supplementary Figure S3.** **Upper and lower 50% percentile: histone modifications change within symbiosis-induced and repressed genes.**

Histone occupation profiles for symbiosis-repressed (n=365) and symbiosis-induced genes (n=366). Genes were divided into upper and lower percentiles based on their median expression fold change. An average profile pattern for each of the histone modifications; H3K27me3 **(A)**, H3K36me3 **(B)**, H3K4me3 **(C)**, H3K27ac **(D)** and H3K9ac **(E)** is shown with respective boxplot comparisons for upper and lower percentiles.

**(F)** Shared TSS dominated histone peaks in symbiosis-repressed and induced genes.


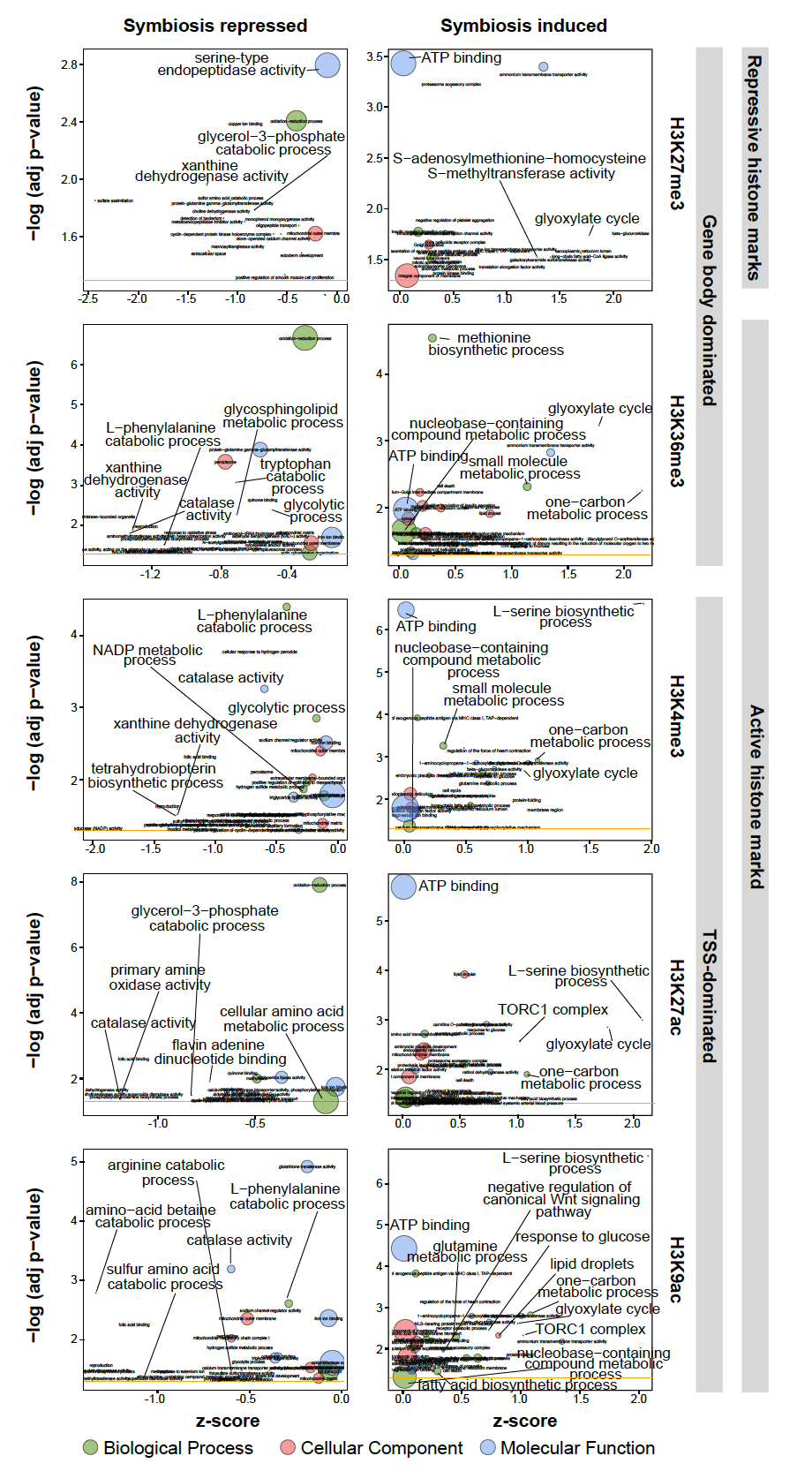


**Supplementary Figure S4.** **Gene Ontology bubble plots.**

Gene ontology (GO): biological process (BP), cellular component (CC) and molecular function (MF) of all five histone modifications (H3K27me3, H3K36me3, H3K4me3, H3K27ac and H3K9ac) associated genes which are repressed and induced in symbiosis. Y-axis is negative of log adjusted p-value, x-axis is z score and area of the circle is the number of genes in the particular category.

**Extended Methods**

Chromatin Immunoprecipitation (ChIP) protocols have been primarily optimized for human, mice, and plant cell studies. The work presented here, and the resulting products, are the first attempts at using ChIP on a symbiotic cnidarian to understand the role of histone modifications in these organisms. In a recent publication (6), a summarized version of the protocol was published. However, the ChIP protocol is sensitive and therefore requires a number of optimization, validation and quality control steps. In the following, we focus on required pre-protocol validations, the optimization and quality control steps taken pre-IP, leading to the final protocol as applied.

**Validation of experimental concept**

**Histone sequence conservation**

While histone modifications are highly conserved across eukaryotes, there has been evidence of histone variants (74). Although these variants do not show altered function of the histone in the nucleosome (i.e., packaging of DNA), a potential change in base pair could hinder the binding of commercial antibodies and, if the target base pair is the modified one, indicate that the modification of interest is not conserved.

The main part of interest is the conservation of the N-terminal tail of histone 3, which carries the modifications investigated in this study. As expected, histone tails are of Aiptasia are highly conserved and align to other plant, animal, and fungi sequences (Supplementary Figure S5). Additionally, we also aligned human histone variants H3.2 and H3.3 to Aiptasia histone models and found continuous conservation of amino acids (98.2% and 100% positives, respectively), indicating that Aiptasia also carries various variant forms of histone 3.

**Supplementary Figure S5. Sequences alignment of histone 3 (H3) across species.** Conservation of amino acid bases is consistent across organisms, particularly across the N-terminal tail (adapted from Li *et al.,* 2018)

The high conservation of the amino acid sequence, particularly on and around sites of interest such as lysine 4 and 9, indicates that the epitope of commercially available antibodies is present and should be detectable. Interestingly, there appears to be a difference in amino acid between organisms at the 98^th^ position; in Exaiptasia and Zebrafish, the sequence carries serine (S) while mice and humans have cysteine (C). Since these positions are in the fold motif of the histone, no modifications occur there. Hence, alterations in this region are not of concern for the purpose of this study.

**Antibody validation through Western blot**

The success of ChIP and its subsequent sequencing is heavily dependent on the antibody quality. Thus, it is important to validate their affinity and sensitivity in order to be used in ChIP studies. Commercial antibodies for H3K4me3 (ab8580, Abcam), H3K27me3 (ab6002, Abcam), H3K27ac (ab4729, Abcam), H3K36me3 (ab9050, Abcam) and H3K9ac (ab4441, Abcam) were validated for use in Exaiptasia. Total protein extraction and western blot was conducted as described in Li *et al.,* (2018). The western blot indicated that all antibodies detected proteins in the expected range, except for H3K4me3, which was highly unspecific (Supplementary Figure S6).

**Supplementary Figure S6. Western blot of histone specific antibodies on total protein content of Aiptasia.** Histone proteins lie in a range of 15-20kDa in size.

These results indicated that effective ChIP could only be expected from 4 out 5 antibodies. However, due to the sensitivity of the ChIP protocol, the final validation of the ChIP protocol only occurs one sequenced data is analyzed.

**ChIP-Seq protocol**

Most ChIP protocols are conducted using individually optimized buffers, depending on the type of cells under investigation. Further research into custom buffers versus kits revealed that the customized steps mostly occur primarily prior to the immunoprecipitation (IP). After antibody incubation, wash and clean up steps follow similar principles across protocols; three washes with increasing salinity followed by DNA clean up and elution. In hopes of streamlining future attempts at ChIP-seq in other cnidarians, especially corals, we opted to optimize pre-IP steps to the point that kits could be confidently used thereafter. The optimization steps described here are based on protocols provided by Valerio Orlando Lab (King Abdullah University of Science and Technology, Saudi Arabia) and Moussa Benhamed Lab (Universite Paris-Saclay, France). After lab optimization steps were established the final protocol was executed.

**Optimization of protocol**

Pre-IP Buffers

Trial and optimization resulted in two pre-IP buffers being used: the fixation buffer and nucleic preparation buffer. The buffers were adapted from pers. comms. *Valerio Orlando and* Schwaiger *et al.* (2014), respectively.

**Fixation buffer**

| Chemical | Final concentration |
| --- | --- |
| 1M Hepes-KOH 7.5 pH | 50mM |
| 5M NaCl | 100mM |
| 0.5M EDTA | 1mM |
| 0.5M EGTA | 0.5mM |
| 37% Formaldehyde | 1% |

Add dH_2_O to fill volume

**Supplementary Table S1: Table showing fixation buffer chemicals and its concentrations.**

**Nucleic preparation buffer**

| Chemical | Final Concentration |
| --- | --- |
| 1M Hepes-KOH 7.5 pH | 50mM |
| 5M NaCl | 140mM |
| 0.5M EDTA | 1mM |
| 50% Glycerol | 10% |
| 10% Triton 100X | 0.25% |
| 100X PIC | 1X |

Add dH_2_O to fill volume

**Supplementary Table S2: Table showing nucleic preparation buffer chemicals and its concentrations.**

**Sonication time**

ChIP-seq requires fragment sizes between 100-600 bp. Ideally, shearing fixed histone-DNA should result in around 200-300bp, since one nucleosome packs around 220-250 bp. Because different types of tissue may behave differently during sonication, it’s important to test the efficiency of time series. We determined that, with 1% formaldehyde fixation for 15 minutes, the optimum sonication time was 15 cycles (15 sec ON, minimum 30 sec cooling) to ensure fragmentation to 200-500 bp.

**ChIP-Seq protocol**

We used the Zymo-Spin ChIP Kit (Zymo Research, Irvine, CA) to conduct histone bound chromatin extraction, with minor adjustments to the manufacturer’s protocol. The experiment was conducted on three biological replicates, each consisting of two symbiotic anemones. The following is a detailed explanation of pre-IP steps modified and adjusted for Aiptasia (Supplementary Figure S7):

1. Anemones were spun down and excess water was removed, followed by a quick rinse in 1x PBST (phosphate-buffered saline with 0.1% triton).
2. Anemones were fixed in formaldehyde buffer containing 1% FA for 15 minutes at room temperature
3. Fixation was stopped by adding 1/20 of the volume 2.5M glycine

*All following steps until elution of DNA should be conducted on ice*

1. Remove solution and wash anemones in cold 1x PBS
2. Suspend anemones in Nucleic preparation buffer and transfer into a douncer for homogenization. Two anemones were crushed at the same time to produce one biological replicate
3. Transfer homogenized tissue into eppendorf and spin for 5min at 500g to collect cellular debri and larger fragments at the bottom of the tube
4. Take the supernatant and transfer to a clean eppendorf.
5. Spin down and collect nuclei in 4°C at 2000g for 10min
6. Resuspend nuclei in Chromatin shearing buffer provided in the kit.
7. Take a sample of your nuclei and dry on glass slide with DAPI staining. Confirm the presence of intact nuclei under the microscope.
8. Sonicate remaining sample for 15 cycles (15 sec ON, 30 sec cooling).
9. Proceed with IP, wash and elute as described in manufacturer’s protocol.

A corresponding input control was maintained for each of the three biological replicates generated. DNA fragment quality and quantity were confirmed using High Sensitivity DNA Reagents (Agilent Technologies, California, United States) on a bioanalyzer. Upon fragment DNA and fragment size validation, ChIP libraries were constructed using NEBNext ChIP-Seq Library Prep Master Mix Set (NEB, Ipswich, MA).

**Supplementary Figure S7. Schematic representation of ChIP-seq protocol using Aiptasia.** Detailed description of each step can be found in section III.2.2.2. Only step 1 to 3 were customized for Aiptasia; step 4 to 5 were conducted as per manufacturer’s protocol.
